# Nitrogen Uptake and Distribution in Different Chinese Cabbage Genotypes under Low Nitrogen Stress

**DOI:** 10.3390/ijms23031573

**Published:** 2022-01-29

**Authors:** Yihui Zhang, Jingjuan Li, Dandan Zhou, Jie Song, Jianwei Gao

**Affiliations:** 1 Shandong Provincial Key Laboratory of Plant Stress, College of Life Science, Shandong Normal University, Jinan 250100, China; zyh_0923@163.com (Y.Z.); lijj0620@163.com (J.L.); zdd6423@163.com (D.Z.); 2 Shandong Branch of National Vegetable Improvement Center, Institute of Vegetables and Flowers, Shandong Academy of Agricultural Science, Jinan 250100, China

**Keywords:** Chinese cabbage, gene expression, nitrogen stress, RNA sequencing

## Abstract

In order to understand the effects of low nitrogen (LN) stress on the growth and development in different genotypes of Chinese cabbage, the L40 genotype with high nitrogen utilization and the L14 genotype with LN utilization were selected as experimental materials. Field experiments and indoor hydroponic methods were used to study the different responses of two Chinese cabbage genotypes to low nitrogen levels. In this study, we also analyzed the genome-wide gene expression profiles of L40 and L14 in response to LN stress by high-throughput RNA sequencing technology. The results reveal that the L40 root system responds better to LN compared with L14. After LN stress, L40 can effectively absorb and transport ${\mathrm{NO}}_{3}^{-}$ and store it in the ground. It is precisely because of this characteristic of the L40 genotype that LN treatment did not have a significant effect on the chlorophyll (Chl) content and net photosynthetic rate (Pn) of the L40 Chinese cabbage compared with the L14 Chinese cabbage. These two different Chinese cabbage genotypes were shown to have differently expressed genes related to nitrate transport, auxin synthesis, and glutamate dehydrogenase synthesis. These genes function in the nitrogen pathway, which are important candidates for understanding the molecular host-response mechanisms to LN stress.

## 1. Introduction

Nitrogen (N) is one of the most essential elements for crop growth and development and is a main component of amino acids, proteins, nucleic acids, Chl, enzymes, and many kinds of vitamins. The ability of plants to absorb N from the soil or from a culture solution mainly depends on the growth and absorption activities of the roots [1]. Plants adapt to low-nitrogen (LN) environments by changing the morphology and structure of their root systems. In an LN environment, the root-to-shoot ratio (RSR) of plants will increase, but its influence on the morphology of the plant roots is more complicated. In Arabidopsis, LN supply inhibits primary root length but increases the number of lateral roots and the root biomass [2,3]. Moreover, studies have shown that auxins play a significant role in the growth and development of plant roots [4]. Nitrilase 2 (NITrase2, NIT2) is a crucial enzyme in the auxin synthesis pathway. Some biotic and abiotic stresses can lead to the differential expression of the NIT2 gene and can regulate the response of plants to stress by regulating the biosynthesis of auxin [5].

Crop roots absorb nitrate from the soil via nitrate transporter 1/peptide transporter (NRT1/PTR) and nitrate transporter 2 family (NRT2). NRT1 belongs to the low-affinity transport system (LATS), while NRT2 belongs to the high-affinity transport system (HATS) [6,7]. Crops can activate different transport systems to effectively absorb ${\mathrm{NO}}_{3}^{-}$ according to the ${\mathrm{NO}}_{3}^{-}$ concentration changes that occur in the environment [8]. The ${\mathrm{NO}}_{3}^{-}$ that is absorbed by crops needs to be reduced to ${\mathrm{NH}}_{4}^{+}$ by nitrate reductase (NR) and nitrite reductase (NiR) before being further absorbed and utilized. After that, ${\mathrm{NH}}_{4}^{+}$ is assimilated into glutamine (Gln) and glutamic acid (Glu) by glutamine synthetase/glutamate synthase (GS/GOGAT) and subsequently synthesizes amino acids. Glutamate dehydrogenase (GDH) catalyzes glutamate synthesis from α-ketoglutarate and ${\mathrm{NH}}_{4}^{+}$, another ${\mathrm{NH}}_{4}^{+}$ assimilation pathway beside the GS/GOGAT pathway, and GDH is found in mitochondria.

In a typical N environment, when the amount of ${\mathrm{NO}}_{3}^{-}$ that is absorbed by crops exceeds their metabolism, the excess ${\mathrm{NO}}_{3}^{-}$ can be stored in the vacuoles. When the external ${\mathrm{NO}}_{3}^{-}$ is gradually depleted, the plant will release the ${\mathrm{NO}}_{3}^{-}$ stored in the vacuole to maintain normal plant growth and metabolism [9]. With the continuous decrease in the external ${\mathrm{NO}}_{3}^{-}$ concentration, the ${\mathrm{NO}}_{3}^{-}$ that is absorbed by the crops decreases, which reduces the inducible expression of *NR* genes and directly affects the ${\mathrm{NO}}_{3}^{-}$ to ${\mathrm{NO}}_{2}^{-}$ reduction process, resulting in insufficient protein synthesis substrates, which is one reason for the decline in the crop biomass. The reduction in the ${\mathrm{NO}}_{3}^{-}$ concentration also changed its distribution pattern in the leaves [10,11], resulting in the amount of N to be used for Chl synthesis being reduced and the Pn of crops being weakened, leading to a decline in crop assimilation, which is another reason for the decrease in crop biomass.

Different crops and varieties of the same crop have significant differences in adaptability to changable N levels. A previous study reported significant genotypic differences in the absorption and utilization of N in wheat [12]. The research results of Jiao et al. also showed that the ability to accumulate N in different potato genotypes is significantly different under various N supply levels [13]. Other reports also showed significant genotypic differences in N uptake and utilization in rice, wheat, maize, potato, etc. [14,15,16,17].

Vegetable fields require a large amount of N fertilizer in order for crops to grow and develop [18]. However, the overuse of chemical fertilizer coupled with long-term continuous cropping and other problems leads to the deterioration of the physical and chemical properties of vegetable planting soil and a decline in vegetable quality year after year, which seriously reduces the economic benefits of farmers [19]. Therefore, reducing the amount of chemical fertilizer input while maintaining the product yield is a crucial problem to be solved in the development of the vegetable industry [20].

Chinese cabbage originated in China, is the largest vegetable crop, and plays an essential role in all-year production, year-round vegetable supply, and market stabilization in China [21]. As a leaf vegetable, Chinese cabbage requires the application of a large amount of N fertilizer to the soil during its growth [22]. In addition, Chinese cabbage is also a vegetable that is prone to ${\mathrm{NO}}_{3}^{-}$ accumulation [23]. In recent years, a great of research has been carried out on the effects of the N application rate and different application methods on the economic characteristics of Chinese cabbage, such as yield and quality. It has been shown that different Chinese cabbage varieties have different nitrogen utilization efficiencies (NUE), and different N fertilizer application rates and methods have essential effects on the economic properties of Chinese cabbage. A higher N application rate cannot improve the yield or quality of Chinese cabbage heads [24,25,26,27,28,29]. A systematic analysis of the genotype differences in 68 Chinese cabbage varieties was performed under two N levels and revealed significant genotype differences in 15 crucial agronomic traits and three NUE traits of Chinese cabbage [30].

In this study, we used two different genotypes of Chinese cabbage to investigate the effects of LN on the accumulation and distribution of nutrients in the roots and shoots of different genotypes at different growth stages. A transcriptome sequence analysis was also performed on the shoots and roots of Chinese cabbage seedlings to understand the expression patterns of the related transcription factors in Chinese cabbage under different N treatments. This will help us to discover the molecular mechanism of the N response in Chinese cabbage, providing a theoretical reference for cultivation.

## 2. Results

### 2.1. Response to LN of Different Chinese Cabbage Varieties

#### 2.1.1. Field Experiment of Chinese Cabbage under Different N Supplying Levels

**Figure 1:**
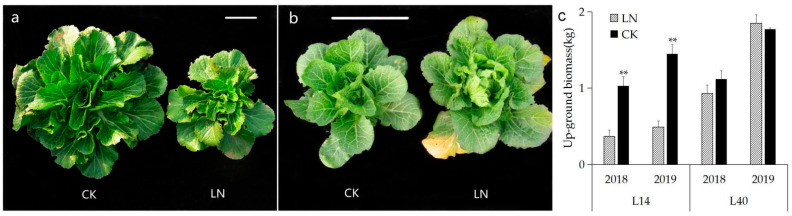
L14 and L40 under different N supply levels in the field. (**a**), L14 under CK and LN conditions. Bar = 5 cm; (**b**), L40 under CK and LN conditions. Bar = 5 cm; (**c**), up-ground biomass (UBM) of Chinese cabbage varieties L14 and L40 under different N supply conditions in the field; ** indicates significant differences at the level of 0.01 according to *t*-test.

The up-ground biomass (UBM) of two Chinese cabbage varieties, L14 and L40, under CK (N: 270 kg/hm^2^) and LN (N: 54 kg/hm^2^) conditions was evaluated based on two years of field experiments. The UBM of the L14 variety under LN conditions significantly reduced, while that of the L40 variety demonstrated no significant differences when compared to conditions in CK in both 2018 and 2019 (Figure 1). The results indicated that L40 was a variety that was tolerant to LN treatment, while L14 was sensitive to LN conditions.

#### 2.1.2. Hydroponic Experiments of Chinese Cabbage under CK and LN Conditions

**Figure 2:**
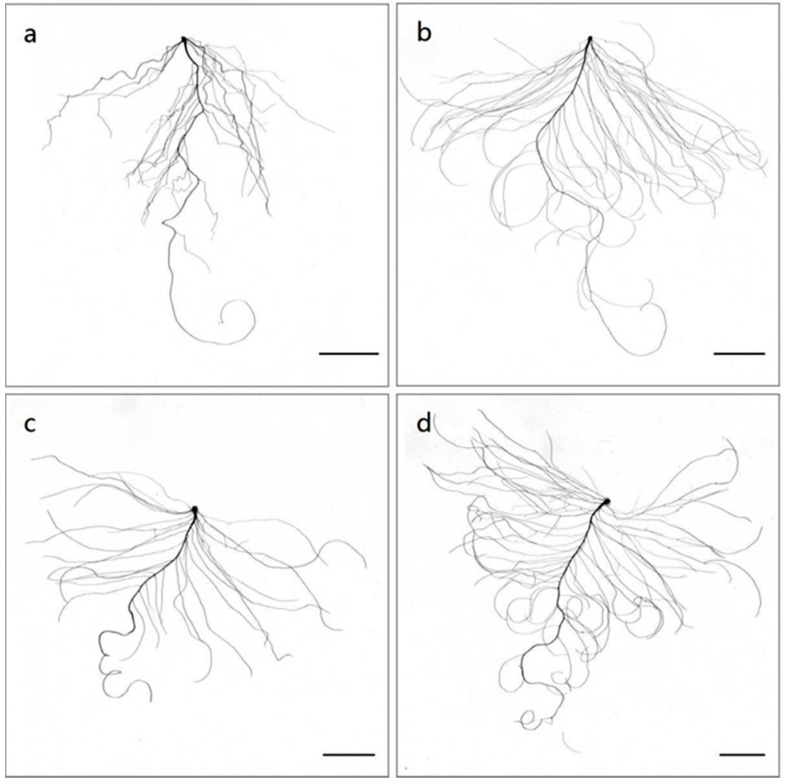
Roots of L14 and L40 under different N supply conditions. (**a**) L14 under CK; (**b**) L14 under LN; (**c**) L40 under CK; (**d**) L40 under LN. Bar = 2 cm.

Hydroponic experiments were performed to further evaluate the sensitivity of L14 and L40 to LN stress in a greenhouse (24 ± 2 °C, 16 h light/8 h dark). Seven days after sowing, shoot and root phenotypes from the L14 and L40 plants under CK (6.0 mM N) and LN (0.2 mM N) treatments were estimated (Figure 2, Table 1). The results showed that the shoot fresh weight (SFW) of the L14 variety was significantly reduced s under LN conditions compared to CK, while that of the L40 variety demonstrated no significant differences under either N supply condition (Table 1). No significant different were determined for the shoot dry weight (SDW) in either variety or between treatments (Table 1). The results implied that the L14 shoot growth was more sensitive to N supply compared to L40.

LN stress led to an increase in certain of the root parameters in both the L14 and L40 varieties, including in the root fresh weight (RFW), root dry weight (RDW), taproot length (TRL), lateral root length (LRL), and root surface area (RSA) (Figure 2, Table 1). However, the increased rates of L14 for most of the root parameters were more significant than those observed for L40, with the exception of TRL (Table 1). For example, the increases in the RFW and RDW for L40 under the LN conditions were significant, while those for L40 demonstrated no significant differences under both N supply conditions. The LRL of L40 under the LN conditions was 2.15 times higher than that under the CK conditions, while that of L40 under the LN conditions was only 1.48 times higher than that under the CK conditions. These results indicate that the root response of L14 to LN treatment was more sensitive than that of L40.

### 2.2. N-RELATED Physiological Indexes under LN Condition in Different Chinese Cabbage Varieties

**Figure 3:**
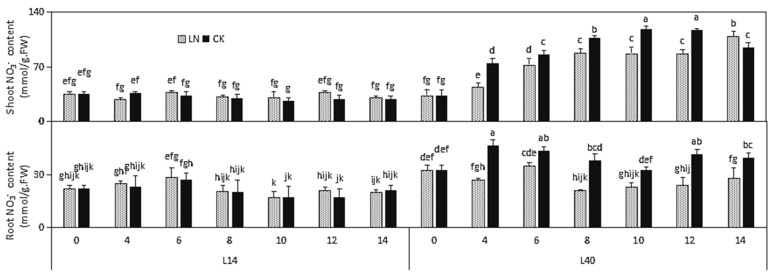
Effects of LN treatment on ${\mathrm{NO}}_{3}^{-}$ content in Chinese cabbage genotypes L14 and L40. Values shown are means ± SE; *n* = 6 (two experiments and three replications of each genotype). Means with different letters are significantly different according to the Duncan test at *p* < 0.05. ${\mathrm{NO}}_{3}^{-}$ content changes under LN stress compared to CK supply are indicated on the bars. The letters “LN” and “CK” indicate whether the N content was low or normal.

The ${\mathrm{NO}}_{3}^{-}$ content in L14 and L40 is listed for both the CK and LN conditions in Figure 3. Under LN stress, no significant differences were determined in the ${\mathrm{NO}}_{3}^{-}$ content in the roots of L14, while a significant reduction in the ${\mathrm{NO}}_{3}^{-}$ content in L40 was observed. However, under LN stress, the ${\mathrm{NO}}_{3}^{-}$ content in the shoots of L40 gradually increased over time. Furthermore, at the two different N levels, L40 had the highest ${\mathrm{NO}}_{3}^{-}$ uptake efficiency at all stages during the growth period, while L14 variety had the lowest ${\mathrm{NO}}_{3}^{-}$ content. This indicates that L40 has a relatively better ability to absorb ${\mathrm{NO}}_{3}^{-}$ under LN stress conditions (Figure 3).

**Figure 4:**
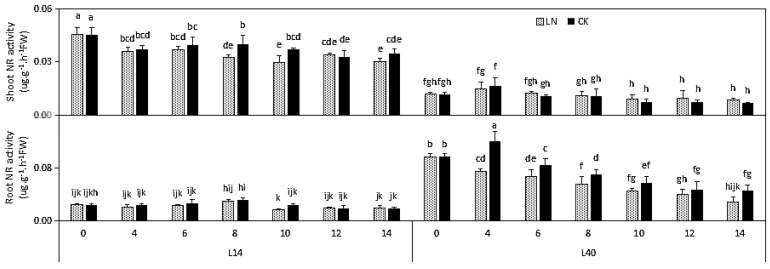
Effects of LN treatment on nitrite reductase (NR) of Chinese cabbage genotypes L14 and L40. Values shown are means ± SE; *n* = 6 (two experiments and three replications of each genotype). Means with different letters are significantly different according to the Duncan test at *p* < 0.05. NR changes under LN stress compared to CK content are indicated on the bars. The letters “LN” and “CK” indicate whether the N content was low or normal.

Significant differences in the NR in the shoots and roots were found among the two varieties. The NR activity in the shoots of L14 was higher than that in the roots, while the opposite trend was found in L40. Moreover, all of the factors (genotype, treatment, and time) and their interactions did not vary significantly. A significant reduction in NR was only observed in the roots of L40 (Figure 4).

**Figure 5:**
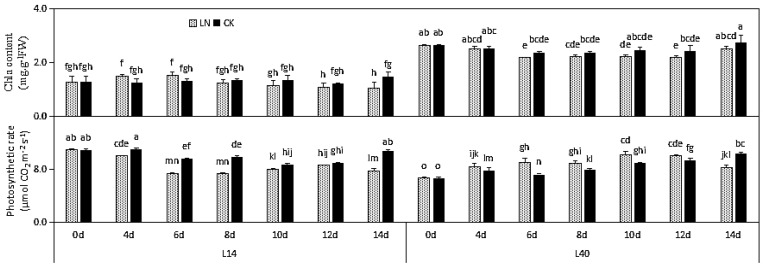
Effects of LN treatment on Chl content and net Pn of Chinese cabbage genotypes L14 and L40. Values shown are means ± SE; n = 6 (two experiments and three replications of each genotype). Means with different letters are significantly different according to the Duncan test at *p* < 0.05. Chl content and net Pn changes under LN stress compared to CK content are indicated on the bars. The letters “LN” and “CK” indicate whether the N content was low or normal.

The Chl content in L40 did not show any significant variations under LN stress, and it was unaffected by time extension. However, the Chl content in L14 appeared to be significantly influenced by LN stress, as well as time extension. In addition, the Chl content in L40 was always higher than that in L14 (Figure 5).

The net photosynthesis rate (Pn) of L14 appeared to be significantly influenced by LN stress and time extension. In comparison, L40 displayed contrasting responses. Under LN stress, the Pn tended to increase compared with CK in L40, and the value under LN stress was lower than that under CK when the experiment terminated (Figure 5).

### 2.3. Transcriptome Analysis of the Effects of LN Stress on Chinese Cabbage

In different treatments, high-throughput transcriptome analysis of plant tissues resulted in 45.1 to 49.6 million raw reads in L14 and L40, respectively (Table 2). Low N (LN) and normal N (CK) treatments were used. All of the treatments had three biological replicates, and the mean assembly percentage to the reference genome ranged from 90.15% to 92.21% for L14 and L40, respectively. The ratios of unique mapped reads were all above 88% (Table 2). Only high-quality reads were used for all of the downstream analyses. In total, 35,527 and 35,282 genes were identified in L14 and L40, respectively. On average, there were 5417 and 5225 novel transcripts in L14 and L40, respectively (Table 2).

**Figure 6:**
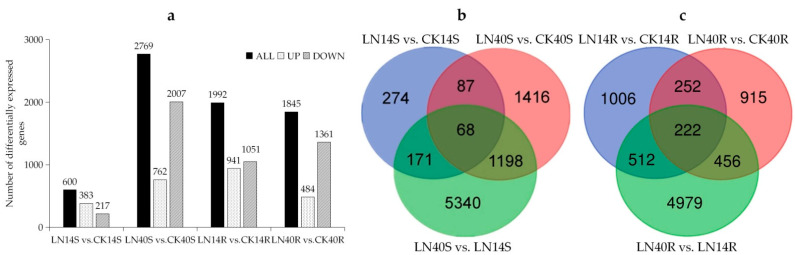
Venn diagrams showing differentially expressed genes (DEGs) in L14 and L40 under LN and CK. (**a**) number of DEGs of in L14 and L40 under LN and CK; (**b**) Venn diagrams of DEGs in the shoots of L14 and L40 when comparing LN vs. CK; and (**c**) Venn diagrams of DEGs in the roots of L14 and L40 when comparing LN vs. CK. Treatment groups are LN14S (L14’shoot-LN), CK14S (L14’ shoot-CK), LN40S (L40’ shoot-LN), CK40S (L40’ shoot-CK), LN14R (L14’ root-LN), CK14R (L14’ root-CK), LN40R (L40’ root-LN), and CK40R (L40’ root-CK).

The differentially expressed genes were identified for each pairwise comparison of L14 and L40 and for the N treatments. The number of common, treatment-specific, and genotype-specific DEGs is illustrated in Figure 6. A total of 23,333 DEGs in response to low N were detected in the shoots of the plant, which included 600 (383 upregulated and 217 down-regulated), 2769 (762 up-regulated and 2007 down-regulated), and 6777 (3003 up-regulated and 3774 down-regulated) DEGs for LN14S (L14-LN shoot) vs. CK14S (L14-CK shoot), LN40S (L40-LN shoot) vs. CK40S (L40-CK shoot), and LN40S vs. LN14S, respectively (Figure 6). On the other hand, in the roots of the plant, the total number of DEGs in response to LN was 21,124, which comprised 1992 (941 up-regulated and 1051 down-regulated), 1845 (484 up-regulated and 2234 down-regulated), and 6169 (2574 up-regulated and 3595 down-regulated) DEGs for LN14R (L14-LN root) vs. CK14R (L14-CK root), LN40R (L40-LN root) vs. CK40R (L40-CK root), and LN40R vs. LN14R, respectively. Overall, the number of DEGs between these two genotypes was higher than it was for other pairwise comparisons.

Furthermore, the number of L14-specific DEGs was 171 (118 up and 53 down), and 512 (241 up and 271 down) for the shoots and roots, respectively (Figure 6). The number of L40-specific DEGs in response to LN was 1198 (368 up and 830 down) and 456 (140 up and 316 down) for the shoots and roots, respectively (Figure 6).

### 2.4. Gene Ontology (GO) and Pathway Analysis for Differentially Expressed Genes under LN Stress

GO enrichment was performed to ascertain the functions of the DEGs and the related biological processes, whereas pathway analysis was conducted to determine the significant and specific plant pathways involved in each N treatment. Combinations of L14 and L40 DEGs from each treatment were used in a singular enrichment analysis (SEA) and plant reactome pathway analysis. In the SEA analysis, the N-responsive DEGs from both L14 and L40 were classified into three domains: “biological process”, “molecular function”, and “cellular component”. There were 76 and 36 significant GO terms identified for L14 and L40, respectively. The “molecular function” had the most significant number of enriched GO terms among the three classified domains, valid for L14 and L40. The “molecular function” domain revealed more “binding” and “activity” GO terms in L14 than in L40. These differences in GO terms showed diversity in response to different N conditions. In addition, the GO enrichment also revealed that the “cellular component” term “photosystem” was only present in L14.

**Figure 7:**
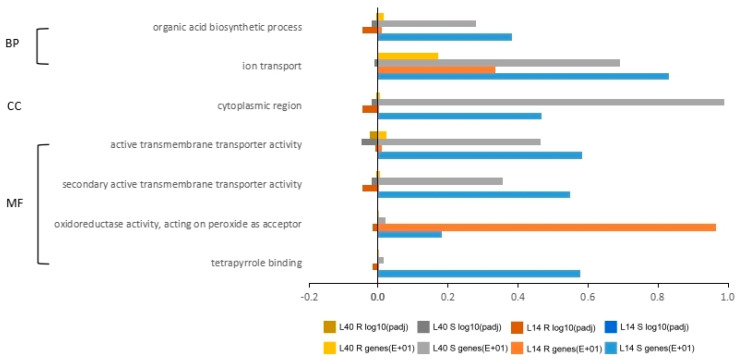
Gene ontology of DEGs from LN treatments in L14 and L40.

The plant reactome pathway analyses of the DEGs showed the major plant pathways that are involved in “environmental information processing”, “metabolism”, and “organismal systems” (Table 3). Most of the DEGs in L14 and L40 were involved in the “metabolism” plant pathway (Figure 7). The primary “metabolism” pathways in L14 and L40 were photosynthesis—antenna proteins and phenylpropanoid biosynthesis. In addition, unique pathways were observed for each genotype: photosynthesis, alanine-aspartate and glutamate metabolism, N metabolism, glucosinolate biosynthesis, circadian plant rhythm, and plant hormone signal transduction; and the MAPK signaling plant pathway, and plant–pathogen interaction was found for L40. These results indicate common primary pathways for both L14 and L40 in response to LN and unique pathways to cope with each N condition.

### 2.5. DEGs Analysis Involved in the N Responsive Process

The N availability in the nutrient solution affected the expression of specific N utilization genes in the roots of L14 and L40, which is essential for N uptake, translocation, and assimilation. Some of these genes were found to be differentially expressed (Table 4).

Nitrilase 2 was differentially expressed in L14 and L40 in LN conditions. However, there was a similar gene regulation pattern in both cultivars.

The N transport/translocation-related genes were low-affinity nitrate transporter genes (*BraA06g009460.3C*, *BraA06g009470.3C*, *BraA08g031180.3C*, and *BraA09g060970.3C*) and high-affinity nitrate transporter genes (*BraA06g005350.3C*, *BraA08g030690.3C*, and *BraA02g005540.3C*). *BraA06g009460.3C* and *BraA06g005350.3C* were differentially expressed in L14 and L40 in LN conditions, and both cultivars showed similar regulation patterns for these genes. *BraA08g030690.3C* was not differentially expressed in the shoots of L14 and L40, regardless of the different N conditions. However, the transporter was differentially expressed in the roots of L14 and L40. *BraA02g005540.3C*, *BraA08g031180.3C*, and *BraA09g060970.3C* only showed significant differences in L40.

Among the N assimilation genes, glutamate synthase 1 (*BraA10g032170.3C*) and glutamine synthetase 2 (*BraA08g009150.3C*) were only present in the roots of L14 under LN conditions, whereas the glutamate dehydrogenase 2 (*BraA03g002960.3C*) showed similar patterns for both cultivars but not in the shoots of L14. Glutamine synthetase 1 (*BraA03g002960.3C*) showed different expression patterns in L14 and L40 under LN conditions. Among the carbon–oxygen lyases genes, beta carbonic anhydrase 4 (*BraA02g019760.3C*) and alpha carbonic anhydrase 3 (*BraA10g032140.3C*) were only present in the roots of L14 under LN conditions, whereas carbonic anhydrase 1 (*BraA01g044810.3C*) was only present in the shoots of L40 under LN conditions. Beta carbonic anhydrase 3 (*BraA07g014010.3C*) and carbonic anhydrase 2 (*BraA02g005650.3C*) showed different expression patterns in L14 and L40 under LN conditions.

### 2.6. Expression Analysis of Essential Genes in N Metabolism Pathway in Response to LN Stress

**Figure 8:**
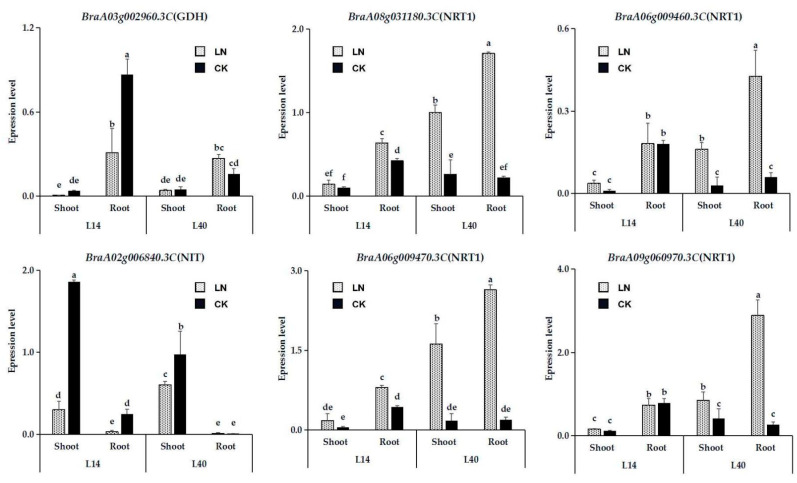
qRT-PCR validation of DEGs from high-throughput sequencing analyses. Relative expression levels were calculated as the reference gene using the formula 2^−ΔCt^. The value indicates the means of three biological replicates ± SE. The letters “LN” and “CK” indicate whether the N content was low or normal. Means with different letters are significantly different according to the Duncan test at *p* < 0.05.

To validate the RNA-sequencing results, six of the DEGs involved in N metabolism pathways, including one *GDH* gene (*BraA03g002960.3C*), one *NIT* gene (*BraA02g006840.3C*), and four NRT1 family genes (*BraA06g009470.3C*, *BraA08g031180.3C*, *BraA06g009460.3C*, and *BraA09g060970.3C*), were selected for real-time quantitative PCR (qRT-PCR) analysis (Figure 8). The results showed that the expression of *BraA03g002960.3C* decreased significantly in L14 under the LN treatment, while the gene expression increased significantly in L40 treated with LN. Although LN treatment significantly inhibited the activity of *BraA02g006840.3C*, the inhibition of *BraA02g006840.3C* in L14 was more significant. Compared to L14, LN significantly induced the expression of four NRT1 family genes in the root of L40, especially *BraA06g009470.3C* and *BraA08g031180.3C*. These results showed that the DEGs had similar expression patterns, indicating that RNA-seq could be used for the gene expression profiling of Chinese cabbage in response to LN conditions.

## 3. Discussion

N is one of the most important elements for plant growth, crop yield, and quality. Thus, the optimization of N fertilizer management along with the adoption of high NUE varieties are the most promising strategies to improve crop yield and environmental protection [31]. Selecting resilient crop varieties has become one of the main objectives to adapt to abiotic stress. In previous studies, response, adaptation, genotype differences, and molecular mechanisms unnder LN stress have been studied comprehensively in Arabidopsis [32], rice [33], maize [34], wheat [35], etc. In Chinese cabbage, yield response and genotype differences have also been studied in LN conditions. As for the molecular mechanisms, detailed and systematic studies are scarce. In this study, two Chinese cabbage varieties, L14 and L40, were investigated under different N supply levels. Both field and hydroponic experiments indicated that L14 is a variety that is sensitive to low N stress, while L40 was shown to be more tolerant. This indicated that L40 may have great potential for yield improvement under LN conditions.

The nutrient absorbing efficiency is one of the fundamental causes of variation in nutrient efficiency [36]. Root morphological and structural properties directly influence the nutrient absorbing efficiency. Plants with longer roots and vigorous lateral roots will have larger root areas through which they can make contact with the medium; therefore, they have a higher potential to take up nutrients [37]. Because of this, most plants extend their root length and increase the root to shoot ratio under nutrient-stressed conditions. The genotype response of the root traits of Chinese cabbage to LN stress was compared in this research. The results showed that with LN stress, most of the root parameters were increased, which is consistent with previous studies [36,37]. However, the response degree was different between varieties. The increased rates of L14 for most of the root parameters were more significant than those found in L40 (Table 1), indicating that the root response of L14 to LN treatment was more sensitive than that of L40. Generally, when the root parameters demonstrate a good response, then it indicates that the plant is better able to adapt to LN conditions [36,37]. However, in our study, the ${\mathrm{NO}}_{3}^{-}$ content and NR activity of L14 were all lower than those of L40. The distribution pattern of the N content in plant leaves can change the Chl content in the leaves, which affects photosynthesis [38]. This study found that LN treatment did not cause a significant decrease in the Chl content and Pn of L40, but it was not the case in L14. This indicates that the roots having a good response to LN stress does not always mean a that the plants under LN conditions have a higher N efficiency.

To mine nitrogen-efficient genes, high-throughput transcriptome analysis was performed, and the DEGs involved in the nitrate metabolic pathway were analyzed in this study. NRT families, including NRT1 and NRT2, are the most prominent nitrate transporter families in plants [6,7,39,40]. After the LN treatment in this study, *four NRT1* genes (*BraA06g009460.3C*, *BraA06g009470.3C*, and *BraA08g031180.3C*, *BraA09g060970.3C*) were identified, which were found to be differently expressed in the roots of L40, while only one *NRT1* (*BraA06g009470.3C*) was identified in L14. For the NRT2 family, three DEGs were detected in the roots, and two DEGs were shown to be more greatly upregulated in the roots of L14 than they were in the roots of L40. Three differently expressed *NRT1* genes were identified in the shoots of Chinese cabbage. *BraA06g009460.3C* was expressed differently in the shoots of both L14 and L40. *BraA06g009470.3C* was only differently expressed in the shoots of L14, while *BraA08g031180.3C* was only expressed differently in L40. No *NRT2* gene was identified to be expressed differently in the shoots of either the L14 or L40 variety. Different *NRT* gene expression patterns in different Chinese cabbage genotypes may provide fine allelic variation for NUE improvement during the cultivation process. The *NRT* genes that were only found to be differently expressed in L40 may be important for LN resistance.

The expression patterns of the genes involved in the nitrate assimilation pathway were also affected by LN stress [41,42,43]. In the study, the four genes related to essential nitrate assimilation (*GDH2*, *GLU1*, *GS2*, and *GSR1*) were identified to be differentially expressed between LN and CK, and the expression patterns were different between L14 and L40 (Table 4). The genotype differences of these genes provided useful information for future molecular mechanisms and NUE improvement studies.

Nitrilase 2 (NITrase2, NIT2), a crucial enzyme in the auxin synthesis pathway, was reported to regulate the response of plants to biotic and abiotic stresses by regulating the auxin biosynthesis [5]. In this study, the expression of the *NIT2* gene *(BraA02g006840.3C)* was up-regulated in the shoots of L40, indicating that LN increased the plant’s own IAA synthesis ability in L40. In conclusion, the genes of the *NIT2* gene may play an important role in the resistance of LN stress for Chinese cabbage.

## 4. Materials and Methods

### 4.1. Test Materials

Under normal N supply (N: 270 kg/ha) and LN (N: 54 kg/ha) conditions, two Chinese cabbage inbred lines with relatively significant differences in terms LN response named L14 (LN sensitive) and L40 (LN tolerant) were screened out based on field agronomic traits and related yield traits. L14 and L40 were acquired from Shandong Academy of Agricultural Sciences, China.

### 4.2. Experimental Design

#### 4.2.1. Field Experiment

L14 and L40 were evaluated at two N levels in the field. The experiment was carried out during the 2018/2019 growing season at the experimental station in Shuangquan Town, Changqing District, Jinan City, Shandong Province (36°39′ N, 116.73° E, 210 m a.s.l.). The experiment consisted of two N treatments (N-normal and N-limiting conditions) and two genotypes with two replications. For the N-normal subplots, 270 kg ha^−1^ of N was added, while in the N-limiting condition, 54 kg ha^−1^ of N was added before seedling transplantation. Each material was randomly planted in one row, with a row spacing of 60 cm and a plant spacing of 50 cm, with 10 plants in each row. There were three replicates for each treatment. During the harvest period, one representative plant for each replicate was selected for trait investigation. Gross weight (GW) was determined at maturity from the mean value of each subplot’s six seedlings.

#### 4.2.2. Solution Culture Experiment

L14 and L40 seeds were previously soaked in 10% sodium hypochlorite for 3 min and were allowed to germinate in culture medium with normal N content (6 mM) in a growth chamber at 24 ± 2 °C (16 h in light, 8 h in darkness) for 13 days. Seedlings of the same size were then transferred in square plastic boxes (three plants in each box) containing a nutrient solution (NS) with normal N content (6 mM) for 24 h in controlled climatic conditions (day/night photoperiod, 16 h/8 h; temperature 26 °C). Then, the seedlings were subsequently grown for 14 d with two N levels (0.2 mM, LN and 6 mM, CK). The nutrient solution was replaced every 4 days. Root and shoot tissue samples were collected from each treatment group after 0, 4, 6, 8, 10, 12, and 14 d to extract the total RNA, to conduct real-time RT-PCR (RT-qPCR), and for analysis and N metabolism. This experiment was repeated three independent times (biological replicates). The specific formulation of the medium is shown in Table 5.

### 4.3. Sampling and Measurement Methods

#### 4.3.1. Investigation of Agronomic Traits

The up-ground biomass was investigated in detail during the maturity period, and the investigation methods and standards were determined based on Appendix 4 of “Chinese Cabbage Breeding” [44].

#### 4.3.2. Root Morphology Analysis

Three Chinese cabbage seedlings from the same growth condition were selected for each treatment, and they were divided into two parts: the shoot and the root. The plant height was measured with a measuring tape. The WinRhizo2007 root measurement system developed by Regent Instruments Canada Inc. (Ottawa, ON, Canada) was used to measure the average diameter of the main root length, root volume, and surface area. The dry weight of the shoot and root parts was measured by the weighing method after drying.

#### 4.3.3. Determination of Related Indexes of Nitrogen Metabolism

Plant materials were sampled after 4 h of normal light conditions. The collected samples were washed with deionized water to remove impurities such as any remaining substrate attached to the plant surface and were wiped dry with absorbent paper. The in vitro method was used to determine the nitrate reductase (NR) activity in the shoots and roots. Moreover, we used ion chromatography to determine the nitrate content in the shoots and roots. Chlorophyll (Chl) content was determined using an extraction method that involved the use of a mixture of ethanol and acetone (1:1) [45]. The net photosynthetic rate (Pn) was measured using a portable photosynthesis system (CIRAS-3, PP-Systems Company, Amesbury, MA, USA).

### 4.4. RNAseq Materials and Methods

#### 4.4.1. RNA Isolation

The total RNA was isolated using Trizol (Invitrogen, Carlsbad, CA, USA). First, the quality of the total RNA was measured using a 1% agarose gel and on a 2100 Bioanalyzer RNA Nanochip (Agilent, Santa Clara, CA, USA). Then, the concentration of the total RNA was measured using a NanoDrop ND-2000 Spectrophotometer (Nano-Drop, Wilmington, DE, USA). Finally, equal quantities of total RNA from three replicates were mixed for subsequent cDNA library construction.

#### 4.4.2. cDNA Library Construction, Sequencing, and Data Processing

cDNA library construction, sequencing, and data processing cDNA libraries were constructed at Novogene (Tianjin, China) according to the manufacturer’s protocol (Illumina Inc., San Diego, CA, USA). The Agilent 2100 Bioanalyzer (Agilent Technologies Inc., Santa Clara, CA, USA) and ABI StepOnePlus Real-Time PCR System (Applied Biosystems, Inc., Foster City, CA, USA) were used to qualify and quantify the sample library, respectively, as per the manufacturer’s instructions. High-throughput sequencing was performed using an IlluminaHiSeq™ 2000 (Illumina Inc., San Diego, CA, USA) at Novogene. Original image data were generated by the sequencer and were transferred into raw sequence data by base calling. After removing empty reads, adapters, and low-quality reads, high-quality clean data were obtained from the raw sequence data. The clean tags were mapped to the *Brassica rapa* L. ssp. *pekinensis* reference genome (http://brassicadb.cn, accessed on 13 December 2021) using SOAPaligner/soap2 [46] with ≤2 mismatches being allowed in the sequence alignment. Unambiguous clean tags were obtained after filtering the tags mapped onto multiple reference genes.

#### 4.4.3. Analysis of Gene Expression Profile of Chinese Cabbage in Response to LN Stress

The gene expression level of all of the samples was normalized according to the RPKM (Reads Per kb per Million reads) method [47]. Differentially expressed genes (DEGs) between the LN-treated groups (L14–LN and L40–LN) and control groups (L14–CK and L40–CK) were screened based on an algorithm developed by Audic and Claverie [48]. We used the threshold “FDR ≤ 0.001 and the absolute value of log2Ratio ≥ 1” to judge the significance of the gene expression difference. All of the data in this study were analyzed using the GO database (http://www.geneontology.org/, accessed on 13 December 2021) and the KEGG database (http://www.genome.jp/kegg/, accessed on 13 December 2021).

#### 4.4.4. Quantitative RT-PCR Analysis

The first-strand cDNA was synthesized using a TOYOBO ReverTra Ace qPCR RT Kit (FSQ-101, TOYOBO, Osaka, Japan). The eight DEGs were selected to validate the expression patterns. Quantitative RT-PCR (qRT-PCR) analysis was performed using a 2× M5 HiPer SYBR Premix EsTaq plus (with Tli RNaseH) (MF787, Mei5bio, Beijing, China) and an IQ5 Real-Time PCR System (BIO-RAD, Hercules, CA, USA). PCR reactions were performed at 95 °C for 30 s followed by 40 reaction cycles (95 °C for 5 s, followed by 60 °C for 20 s). The gene-specific primers were designed by Primer Premier 5.0, as shown in Table 6.

#### 4.4.5. Data Processing and Analysis

Excel 2016 and SPSS 10.0 statistical software were used for drawing and for statistical analysis, as described by Anwar et al., 2018. The Duncan method was used for multiple comparisons. The means ± standard deviation represents the data in the chart.

## 5. Conclusions

In conclusion, the up-ground biomass of L14 was greatly reduced by low N stress in both the field and hydroponic experiments, while no significant differences were observed in L40 when compared with the Chinese cabbage grown in CK conditions. LN stress increased most of the root parameters in both L14 and L40, but the degrees in L14 were more significant than those L40, with the exception of TRL. LN treatment did not cause a significant decrease in the Chl content and Pn in L40, while those of L14 decreased significantly. The results indicated that L14 was sensitive to low N stress, while L40 was more tolerant. L40 may have great potential for yield improvement under LN conditions.

According to high-throughput transcriptome analysis, totals of 23,333 and 21,124 DEGs were detected in the shoots and roots, respectively, in response to low N. Out of these DEGs, 17 genes involved in the nitrate metabolic pathways, including N transport, N assimilation, photosynthesis, and auxin synthesis, were identified to be expressed differently between LN and CK, and the expression patterns were different between genotypes. These genes are the most important candidates for further studies. This study will help us to understand the molecular mechanism of the N response in Chinese cabbage, thus providing a theoretical reference to improve NUE cultivation.

## Figures and Tables

**Table 1:** Shoot and root phenotypes of L14 and L40 under different N supply conditions.

Part	Trait	L14		L40	
		LN	CK	LN	CK
Shoot	SFW(g plant^−1^)	0.75 ± 0.08 b	0.93 ± 0.39 a	0.72 ± 0.07 b	0.74 ± 0.09 b
	SDW(g plant^−1^)	0.027 ± 0.001 a	0.034 ± 0.007 a	0.027 ± 0.001 a	0.026 ± 0.005 a
Root	RFW(g plant^−1^)	0.097 ± 0.009 a	0.064 ± 0.007 b	0.103 ± 0.009 a	0.087 ± 0.009 ab
	RDW(g plant^−1^)	0.0063 ± 0.0002 a	0.0044 ± 0.0004 b	0.0054 ± 0.0006 ab	0.0047 ± 0.0005 b
	TRL(cm plant^−1^)	19.23 ± 1.58 a	18.67 ± 2.08 ab	14.87 ± 0.91 b	11.27 ± 2.61 c
	LRL(cm plant^−1^)	363.41 ± 12.47 a	169.1 ± 6.77 c	279.78 ± 12.06 b	188.41 ± 8.95 c
	RSA(cm^2^ plant^−1^)	18.42 ± 1.86 a	10.41 ± 1.36 b	17.26 ± 0.97 a	12.15 ± 1.62 b

Values shown are means ± SE. Means ± SE with different letters are significantly different according to the Duncan test at *p* < 0.05.

**Table 2:** Statistics of categorization and abundance of tags generated from the eight cDNA libraries for DEG analysis.

Sample	Total Raw Reads	Total Clean Reads	Q20 Percentage	Total Map	Unique Map	Total Genes	Novel Transcript
CK14S	46,383,112	45,027,680	98.45	92.21%	89.81%	29,776	5324
CK14R	47,421,702	45,802,951	98.31	90.15%	88.41%	29,771	5319
LN14S	46,323,061	45,363,247	98.39	91.91%	89.92%	29,585	5133
LN14R	49,639,339	48,482,294	98.31	90.25%	88.27%	30,335	5883
CK40S	47,193,171	46,107,124	98.43	91.90%	89.03%	29,167	4786
CK40R	46,282,418	45,051,759	98.30	90.51%	88.68%	29,308	4927
LN40S	46,018,595	44,930,999	98.39	91.08%	89.79%	29,951	5570
LN40R	45,126,988	44,067,133	98.28	90.53%	88.68%	30,000	5619

**Table 3:** Pathway classification of differentially expressed genes in L14 and L40 (*p*-value ≤ 0.05).

KEGGID	Description	LN14L vs. CK14L		LN14R vs. CK14R		LN40L vs. CK40L		LN40R vs. CK40R	
		*p*-Value	GeneRatio	*p*-Value	GeneRatio	*p*-Value	GeneRatio	*p*-Value	GeneRatio
ath00053	Ascorbate and aldarate metabolism	0.006	4/62	0		0		0	
ath00195	Photosynthesis	0.000	10/62	0		0		0	
ath00196	Photosynthesis—antenna proteins	0.000	9/62	0.034	5/191	0.000	10/642	0	
ath00220	Arginine biosynthesis	0		0.012	3/191	0		0	
ath00250	Alanine, aspartate and glutamate metabolism	0		0.000	9/191	0		0	
ath00280	Valine, leucine and isoleucine degradation	0		0.006	7/191	0		0	
ath00350	Tyrosine metabolism	0		0.030	5/191	0		0	
ath00380	Tryptophan metabolism	0		0		0.004	14/642	0	
ath00400	Phenylalanine, tyrosine and tryptophan biosynthesis	0		0.028	6/191	0		0.042	4/111
ath00460	Cyanoamino acid metabolism	0		0.046	5/191	0		0.036	4/111
ath00640	Propanoate metabolism	0		0.044	4/191	0		0	
ath00730	Thiamine metabolism	0.038	2/62	0		0		0	
ath00860	Porphyrin and chlorophyll metabolism	0.035	3/62	0		0		0	
ath00904	Diterpenoid biosynthesis	0		0		0		0.005	3/111
ath00906	Carotenoid biosynthesis	0		0		0		0.007	4/111
ath00908	Zeatin biosynthesis	0		0.013	4/191	0		0	
ath00910	Nitrogen metabolism	0		0.000	10/191	0		0	
ath00920	Sulfur metabolism	0		0.006	6/191	0.008	11/642	0	
ath00940	Phenylpropanoid biosynthesis	0		0.000	21/191	0		0.000	13/111
ath00960	Tropane, piperidine and pyridine alkaloid biosynthesis	0		0.012	5/191	0		0	
ath00966	Glucosinolate biosynthesis	0		0.001	5/191	0.026	6/642	0	
ath04016	MAPK signaling pathway—plant	0		0		0.000	32/642	0.000	12/111
ath04075	Plant hormone signal transduction	0		0		0		0.002	15/111
ath04130	SNARE interactions in vesicular transport	0		0		0.008	11/642	0	
ath04626	Plant-pathogen interaction	0		0.018	13/191	0.034	31/642	0.000	14/111
ath04712	Circadian rhythm—plant	0.001	4/62	0		0		0	

**Table 4:** Number of identified DEGs related to LN stress response.

Function	Gene Name	Gene ID	Chr.	Start	End	log2 Ratio (LN/CK)			
						Root		Shoot	
						L14	L40	L14	L40
Nitrate transport	NRT1	*BraA06g009460.3C*	A06	5,163,603	5,163,896		2.43	1.78	1.32
	NRT1	*BraA06g009470.3C*	A06	5,167,089	5,168,324	1.42	1.94	1.73	
	NRT1	*BraA08g031180.3C*	A08	20,956,829	20,960,518		1.13		1.55
	NRT1	*BraA09g060970.3C*	A09	42,614,336	42,618,089		1.31		
	NRT2.1	*BraA06g005350.3C*	A06	3,179,020	3,180,823	8.16	3.34		
	NRT2.5	*BraA08g030690.3C*	A08	20,761,898	20,763,666	8.37			
	NRT2.7	*BraA02g005540.3C*	A02	2,623,331	2,624,856		−2.01		
Nitrate assimilation	GDH2	*BraA03g002960.3C*	A03	1,321,750	1,323,677	−2.11	−1.15		−1.03
	GLU1	*BraA10g032170.3C*	A10	19,964,582	19,972,116	1.02			
	GS2	*BraA08g009150.3C*	A08	8,134,274	8,136,484	1.62			
	GSR1	*BraA04g010460.3C*	A04	8,265,796	8,268,190	1.03			1.48
Photosynthesis	ACA3	*BraA10g032140.3C*	A10	19,954,817	19,956,218	−2.45			
	BCA3	*BraA07g014010.3C*	A07	12,918,461	12,920,370	4.14		−1.34	3.42
	BCA4	*BraA02g019760.3C*	A02	11,362,629	11,365,101	1.21			
	CA1	*BraA01g044810.3C*	A01	29,518,958	29,521,894				−1.41
	CA2	*BraA02g005650.3C*	A02	2,674,642	2,677,016	4.37	−3.24		
Auxin synthesis	NIT2	*BraA02g006840.3C*	A02	3,245,216	3,246,972				1.28

**Table 5:** The hydroponic nutrient solution content for each treatment.

Chemical Element	Nutrient Solution	Treat	
		CK(6.0 mM)	LN(0.2 mM)
ME	KNO_3_	3 mM	0.1 mM
	KCl	0.0 mM	3 mM
	KH_2_PO_4_	1.5 mM	1.5 mM
	K_2_HPO_4_	1.5 mM	1.5 mM
	(NH_4_)SO_4_	0.75 mM	25 µM
	MgCl_2_	1 mM	1 mM
	K_2_SO_4_	2 mM	2 mM
	Ca(NO_3_)_2_	0.75 mM	0.75mM
	CaCl_2_	3.25 mM	3.25 mM
Fe	Na_2_Fe-EDTA	40 µM	40 µM
TE	H_3_BO_3_	60 µM	60 µM
	MnSO_4_	14 µM	14 µM
	ZnSO_4_	1 µM	1 µM
	CuSO_4_	0.6 µM	0.6 µM
	NiCl_2_	0.4 µM	0.4 µM
	H_2_MoO_4_	0.3 µM	0.3 µM
	CoCl_2_	20 nM	20 nM

Note: ME: macroelement; Fe: Ferrum; TE: trace element.

**Table 6:** Sequences of primers used in the study.

Genes	Forward Primer	Reverse Primer
*BraA08g033360.3C* (Bra-Action)	ATACCAGGCTTGAGCATACCG	GCCAAAGAGGCCATCAGACAA
*BraA06g009460.3C*	ATTGAGCACATTGGCGTTAGGA	ACCACTTGGAGAAAACGAGGAAA
*BraA06g009470.3C*	CTTCCTGAAACAAAATCTCAAACCC	AAGCCACCGAGGAGACAGAGC
*BraA08g031180.3C*	TTATTGCGGCGGAAGGCG	CATCGGTTAGTGTTGAAAGTGTCCA
*BraA09g032690.3C*	ATTTCCTCCTGGCTGCCTTGA	TGGGTCGCTCCTCGTAGTCGT
*BraA02g006840.3*C	CGGTGCTGCTATTTGCTG	CTTCGTGTTGCTCTGGGT
*BraA03g002960.3C*	TGAAACACAAAGAGGCAA	ATGGAGCAACAGTTCGTC

## Data Availability

Not applicable.
